# Effects of teaching experience and culture on choral directors’ descriptions of choral tone

**DOI:** 10.1371/journal.pone.0256587

**Published:** 2021-12-07

**Authors:** Emily Y. Frizzell, Leah Cathryn Windsor

**Affiliations:** 1 Rudi E. Scheidt School of Music, University of Memphis, Memphis, Tennessee, United States of America; 2 Department of English and Institute for Intelligent Systems, University of Memphis, Memphis, Tennessee, United States of America; University of California, Los Angeles, UNITED STATES

## Abstract

In this study we examine the effects of experience and culture on choral teachers’ description of choral tone across a range of genres. What does a “good” choral music performance sound like? Is there an objective standard of performance excellence, or is beauty in the eye of the beholder? In teacher preparation programs, choral directors in the United States have been taught to identify and teach particular, culturally-bounded standards of choral tone in their students. Choral directors evaluate their students’ voices along two dimensions: health and appropriateness. They discern and describe whether the student’s musical instrument—their voice—is producing sound in a healthy and non-damaging way. They also judge whether the style of their sound is appropriate for the music they are singing. However, teacher preparation programs do not provide common standards or lexicon for describing tone. This may increase implicit bias of individual directors, and inadvertently exacerbate ethnocentrism and harm students’ self-perception. Using a computational text analysis approach, we evaluate the content of open-ended survey responses from teachers, finding that the language used to describe and rate choral performance varies by experience, and by the choral selection (e.g., whether it is a traditional Western or non-Western song). We suggest that regularizing the terminology and providing common training through professional organizations can minimize potential bias and generate more systematic, precise use of qualitative descriptors of health and appropriateness, which will benefit students and teachers.

## Introduction

A typical scholastic choir may experience a situation like this at a performance adjudication event: *A high school choir proudly shows off the product of their semester of diligent work*. *They perform three pieces in varied styles for the reputable choral music educators serving as judges*: *“Domine Fili Unigenite” by Palestrina; “The Seal Lullaby” by Eric Whitacre; and “Kaval Sviri”by Petar Lyondev*. *Leaving the performance*, *the choir feels confident in their performance*, *knowing they remembered their training and sang to the best of their ability*.

In their feedback, Judge 1 complimented their supported tone in their performance of “Kaval Sviri,” a Bulgarian folksong. Judge 2 raved about their attention to detail, praising them for their authentic-sounding performance that showed respect to the culture of the same piece. Judge 3 commented that when singing the Bulgarian piece, the singers’ tone was strident, heavy, and unhealthy and that they shouldn’t sing in such a style. The choir that earlier felt confident in their performance is now disheartened and confused.

Choir directors are encouraged to introduce diverse musical styles into the choral repertoire [1,2] but, when doing so, may encounter conflicting assessment feedback as described in this scenario. To provide a thorough education to their students, choral music educators require a comprehensive understanding of choral techniques appropriate to each style they teach. Traditionally, American scholastic choral programs have prioritized music from the Western European canon [1,3–7]. The inclusion of more diverse choral literature from across the world requires that teachers know culturally specific techniques to be able to teach them to their students. While teachers are increasingly concerned with authenticity in their representation of non-Western choral music [7–9], many choral music teacher preparation programs lag in curricular requirements that would support this goal [2–6,10]. This situation poses a conundrum: while choral music educators may be eager to diversify the choral music they teach, they may not have requisite proficiency and training to convey authentic performance techniques to their students or to evaluate the authenticity of their students’ performances [2–6].

To gain insight into the implications of these plausible deficiencies, we fielded a survey experiment of scholastic choir directors to evaluate how they interpret one aspect of choral music performance, choral tone quality, across a variety of musical styles, and how their perceptions may be influenced by their years of teaching experience and their cultural perspective. This paper proceeds as follows: we first provide context in a review of literature for factors relating to choral tone as it is widely taught to American preservice choral music educators. We then theorize about how years of teaching experience and cultural constructs shape how choir directors evaluate performances. We then describe the procedures and methodology of our analyses of choral directors’ descriptions of choral tone. Then, we deliver our results, including the significant differences between choral directors’ descriptions of choral tone. We end with a discussion of how knowledge of choral directors’ descriptions of choral tone can influence the future of teacher training and professional development in choral music education.

## Literature review

### Choral tone preference

Choral directors assess choral tone along two dimensions: if the sound is produced in a healthy and non-damaging way [11–14] and if the style of the sound is appropriate for the music the choir is singing [15–21]. Research has demonstrated that quality ratings may differ between scholastic choral director experience groups, with preservice teachers rating performance quality lower than more experienced teachers [14,22,23]. Additionally, music preference may influence ratings of music performances [24,25]. Furthermore, music preference may evolve with exposure to a variety of musical styles, but is dependent on several factors, including systemic and cultural influences [19,26,27].

### Current status of the field: A lack of universal lexicon

There is no objective standard for choral tone for K-12 choir teachers to use as a guide [14,28,29]. Even though national and state departments of education provide content- and grade-specific standards, they leave much room for interpretation. These standards mandate the implementation of a “varied repertoire of music representing diverse cultures,” but do not provide activities or pedagogy [30]. In “On the Art of Singing,” Miller writes that the “language describing breath management, laryngeal function, and resonator response should not be inventions of the moment. These are functions common to all who breathe and phonate, and can be described through precise language [31].” Yet in practice, choir teachers are not given universal precise language to convey to students how to produce an ideal tone. Experiencing vocal tone is subjective [28,29], and as Miller notes, “Managing one’s own instrument and describing how it feels to oneself is not sufficient for the instruction of others [12].” As such, choir teachers are challenged to teach using imprecise descriptions of choral tone, drawing from their own vocal experiences, while striving to adhere to the subjective quality standards of potential adjudicators.

As McKinney (2005) notes, “A necessary prerequisite of establishing good phonatory habits is for the singer or speaker to possess a valid concept of good vocal sound” [11]. Good vocal sound is described as: freely produced; pleasant to listen to; loud enough to be heard easily; rich, ringing, and resonant; energy flows smoothly from note to note; consistently produced; vibrant, dynamic, and alive; flexibly expressive [11]. On the other hand, poor vocal sound is described as: constricted, forced, or strained; strident or rasping; too loud, resembling shouting or yelling; hoarse; breathy; weak, colorless, or devitalized; inconsistently produced; shaky or wobbly [11]. Choral beauty is subjective, in the ear of the beholder.

### Music across cultures: A diverse student population

Culture can be difficult to define in the modern scholastic choir. The terrain of Western choral music is well-worn with common performances of pieces like “Kaval Sviri.” However, scholastic choirs and university choral music education programs are at the frontier of addressing how to meaningfully integrate more diverse repertoire into the modern American choral program [2,5,7,8,10,32,33]. This increase in diverse repertoire is in response to the changing composition of student populations, greater exposure to non-Western cultures, and increased exposure to non-Western music through popular culture.

The student body present in choral music classes is more diverse today than in previous generations [1,34,35]. Students may be recent immigrants or speak English as a second language; Gluszek and Dovidio found non-native speakers who spoke with an accent experienced a feeling of a lack of belonging [36]. Others have investigated the ways in which non-native accents can affect value judgments from native speakers, including the perception that there are good and bad accents, and right and wrong pronunciations of language [37,38]. It stands to reason that the judgments of singing, as a melodic production of language, may also be subject to these kinds of inherent biases as well [24,25,39].

### Western choral tradition in a globalized world

In the Western choral tradition, particularly in the United States, choral directors are taught to sing and to teach students to sing with a lowered larynx, rounded lips, tall vowels, and other similar parameters in order to facilitate a clear, resonant sound [11,31,40]. Additionally, choir directors place priority in maintaining blend and balance across the ensemble [13,41]. Techniques used to teach blend and balance do not necessarily translate to non-Western styles of choral music [40,42]. Additionally, cultures vary in choral expectations regarding volume, physical posture, and artistic vocal technique such as ululation, trilling, yodeling, and vibrato [43].

The majority of licensed choir teachers graduate college with a degree in choral music education. University degree plans in choral music education may meet accreditation standards set by the National Association of Schools of Music (NASM), but may comprise different courses and requirements within those standards. Therefore, for better or for worse, not all degrees are created equal. For example, in regard to content, repertories, and methods, the NASM Handbook (2019–2020) states:

With regard to specifics, music has a long history, many repertories, multiple connections with cultures, and numerous successful methodologies. Content in and study of these areas is vast and growing. Each music unit is responsible for choosing among these materials and approaches when establishing basic requirements consistent with NASM standards and the expectations of the institution [44].

While these liberties allow for relevant, practical, and accessible higher education in music, they leave enough flexibility that certain student experiences, like exposure to multicultural content, may be limited [5,10,45]. In regard to tone quality, students do not typically take a singular course on the subject, but learn through applied voice lessons and choral ensembles. Curricula and literature for these courses are selected by the instructors. Instructors consider many factors when selecting literature, including student performance ability, context of the performance, variety of style, tempo, language, and more [13,41]. Although variety within program literature is, theoretically, prioritized, many instructors select primarily Western art music [5,7,10].

Since music programmed by K-12 and university choir directors is largely Western in derivation [3,5,10], it follows that the tone required for those musics receives more time and attention than tone from any other culture or practice. Furthermore, courses in subjects such as ethnomusicology, vocal pedagogy, and choral literature may be offered at the majority of universities [3,46,47], but are not universally required of students in choral music education programs [3]. Therefore, students may be graduating with limited knowledge in non-Western music practices such as multicultural music traditions, diverse tone production practices, and varied repertoire because of institutional and curricular biases and deficits [3,4,45].

Prioritizing Western choral tone techniques results in cohorts of preservice music educators unfamiliar with practices in multicultural music [3–5,7,10], particularly practices in healthy and appropriate tone production [48]. The lack of exposure may also affect their confidence to try new non-Western pieces for two key reasons. First, choral director unfamiliarity with healthy tone production across styles may result in the inability to detect student vocal health problems across styles [40]. Second, choral director unfamiliarity with appropriate tone production across styles may result in the inability to detect inappropriate tone production across styles, potentially leading to offensive performance practice [10,32,40,48,49]. Diversifying the curriculum can empower preservice choral music educators to help the choral community preserve distinctive cultural traditions.

### Years of teaching experience

Exposure to non-Western musical styles may be endogenous with teacher experience. Research shows choral directors with more experience rate choral performances overall higher than choral directors with less experience, suggesting choral directors’ perceptions change with years of experience [14,22,23]. The gap between the theory—what choral directors learn in teacher preparation programs—and the practice—what choral directors learn during their years teaching, grows with years of teaching experience. Additionally, teachers may have different perceptions about, and exposure to, music from non-Western traditions. They may utilize technology differently to access, research, and interpret music from outside the canonical teachings. They may vary in fluency with social media and exposure to global culture through their connections to diverse populations and through their online connections.

Choral directors have wide berth to use their own judgments and experience to convey the essence of proper vocal sound to students [12,31]. The lack of objective standards and reliance on subjective judgment introduces strong variation in judging even a singular genre of Western choral style music, and is amplified even more when teachers are asked to evaluate musical styles and performance practices from a variety of global genres. To frame these competing demands, we rely on the theories of implicit bias, cultural exposure, and the gap between theory and practice.

## Theory

### Two cultures: Teaching experience and globalization

Howell (1982) generated a framework for cultural competence that incorporates elements of awareness and proficiency [50]. We apply this framework to both cultural awareness of musical styles and to the culture of classroom experience as shown in Table 1. We theorize that there is an inverse relationship between years of teaching experience and cultural fluency in different musical genres from throughout the world. As Pettigrew and Tropp (2005) found, increased intercultural exposure and contact reduces bias among people from different backgrounds [51]. In a review of bias reduction strategies, Paluck and Green also found that cross-cultural contact, peer interaction, and cooperative learning can help to reduce cross-cultural bias through the diffusion of social norms and the increased proficiency in understanding different perspectives [52].

**Table 1:** Competence, culture, and experience.

	Pre-service	Novice	Intermediate	Experienced
**Years (mean, max)**	0, 1 Years	7, 17 Years	16, 31 Years	14, 40 Years
**Unconscious incompetence**				
**Teaching**	X			
**Culture**				X
**Conscious incompetence**				
**Teaching**		X		
**Culture**			X	
**Conscious competence**				
**Teaching**			X	
**Culture**		X		
**Unconscious competence**				
**Teaching**				X
**Culture**	X			

Theories of psychological development suggest that adolescents and young adults may be more neuro-biologically primed to integrate new information [53]. Further, communication accommodation theory suggests that social identity is reinforced through repeated interaction [54], and that members of linguistic communities increasingly adopt similar language over time [55]. Similarly, members of particular generations innovate and use new terms that mark their group membership, such as “awesome,” “groovy,” “rad,” and “fleek.” The usage of these new terms may be counter-balanced in the classroom by pre-service and novice teachers because of over-reliance on terminology learned by rote, such as words used to describe the health and appropriateness of choral music. Intermediate and experienced choir teachers are likely to use terminology that is common among their peer groups, solidified and reinforced by years of exposure.

In Howell’s model, the first stage is unconscious incompetence, where individuals are unaware that they lack a skill set. In this stage, they may adhere rigidly to rules that govern their current perspective and behavior. In our construct, we associate unconscious incompetence with pre-service music teachers, who have limited teaching experience. These future music educators are likely to have a mastery of theoretical rules for teaching music and, specific to our study, the production of choral tone. Unconscious incompetence may also align with our sample’s most experienced teachers’ cultural perspectives. It is possible that the most experienced teachers may have had less training early in their careers in a variety of genres and their respective choral tone production techniques than our more recent college graduates. These experienced teachers have likely reinforced their practices over time and may rely less on outside resources, such as YouTube or international journals, when preparing their lessons. Therefore, they may be unaware if they lack certain skills regarding cultural differences in health and appropriateness of choral tone.

The next stage is conscious incompetence, where, in this case, teachers with some experience (“novices”) are cognizant of the skills they lack in the classroom, and are eager to bridge the gaps between theory and practice in teaching. This stage for teachers marks the onset of self-awareness, differentiation from textbook definitions when teaching, and the onset of the development of one’s own teaching style. This stage also corresponds to cultural awareness of teachers who now have enough classroom experience, and life experience, to have encountered, researched, and taught music from a variety of other cultures. For these “intermediate” teachers, this is the “cultural smorgasbord” phase, where they are secure in their classroom management and instruction, and begin to explore different genres of music in earnest, becoming more entrenched in what they know.

Next in Howell’s taxonomy of cultural awareness is conscious competence. At this stage, people perform tasks with competency, but also with self-awareness. As it relates to teaching, intermediate teachers may reflexively reflect on their teaching strategies through active self-critique and adapt their instruction accordingly. Novice teachers may also exhibit conscious competence, but as it relates to culture. Today’s novice teachers have likely been exposed to a variety of musical genres as students. As instructors, they likely keep their theoretical knowledge of vocal health at the forefront of consideration, while attempting to maintain an open-minded and individually-researched approach to stylistic appropriateness of choral tone.

The final stage is unconscious competence, where people are fluent in a behavior and do not have to deliberately process or reflect on their actions. The most experienced teachers demonstrate unconscious competence in their teaching. They have heard so many voices and instructed so many students that addressing and correcting vocal techniques is second nature to them. Unconscious competence also corresponds to preservice teachers who may accurately emulate the vocal styles of musical genres outside the Western choral tradition without considering the health or appropriateness of the selection. Today’s preservice teachers are likely to be culturally proficient and fluent as a result of having grown up in an era of globalism in music through unfettered access and exposure online to film and music. As diversity within the United States grows, choir students today are more likely to personally know people from other countries and cultures, theoretically increasing acceptance of a wider variety of performance practices regarding choral tone.

From this discussion we generate the following hypotheses:

*Hypothesis 1a*: Choral directors with more years’ experience should rate performances as healthier than choral directors with less experience.
*Hypothesis 1b*: Choral directors with less experience should rate performances as more appropriate than choral directors with more experience.
*Hypothesis 2*: Choral directors should rate culturally familiar performances as healthier and more appropriate than those different from their experience and training.

### Health and appropriateness

At present, the lexical landscape of descriptors for vocal tone consists of terms related to color (bright, dark), shape (tall, round), resonance (covered, open), volume (yelling), texture (smooth), and strength (forced, devitalized). Choral teachers are not provided with a separate list of terms to describe appropriate, or inappropriate, vocal sound, although terms for poor vocal sound tend to overlap with unhealthy practices [43]. Given the lack of terminological distinction for vocal tone, it is possible that choral directors mislabel or misclassify unfamiliar musical performances—those from outside their traditions, culture, and experience—as unhealthy or stylistically inappropriate.

The language that choral directors use can also reveal information about their status or experience. People with higher status or more experience tend to use fewer words to express themselves, whereas people with lower status or less experience tend to use more words to express themselves [56]. This includes more descriptors such as adjectives and adverbs, and more positive and negative emotion words to qualify their descriptions. Following this, we would expect that newer teachers would be more verbose in their descriptions of vocal performances than their more senior, experienced peers. Because of this, we may be able to learn more about the terminology that practitioners use to describe various types of tone production they encounter in choral performances.

From this discussion, we generate the following hypotheses:

*Hypothesis 3a*: Choral directors will use more descriptors (adjectives, adverbs, negations), more positive emotion, and less negative emotion with performances they find healthier and more appropriate.
*Hypothesis 3b*: Choral directors will use more discrepancy terms (should, ought) for performances they find less healthy and appropriate.
*Hypothesis 3c*: Choral directors with more experience will use fewer words than those with less experience.

We now turn to a discussion of the data, methods, and results of our survey experiment.

## Method

The methods and analysis involve several steps to transform the open-ended survey responses into numerical values that we can compare and analyze. We first provide the descriptive elements of the survey, followed by a detailed presentation of each of the approaches we use to evaluate choir directors’ language. Each of the methodological approaches, such as LIWC and LDA (which we describe in detail subsequently in this section), represent an interim step in the analytical process.

We distributed the survey electronically to members of national, state, and regional choral director associations and Facebook groups for choir directors. The survey instrument is called The Choral Tone Assessment (CTA). It was created using Qualtrics^™^. Participants were asked to provide demographic information such as age, years teaching experience, race, and sex.

### Procedures

We sent participants the CTA survey electronically. The survey was embedded with instructions, audio recordings, and test items. Instructions were to listen to each recording and answer five Likert-scale items and one open-response item for each. No length requirement was given on the open-response item. We estimated the survey completion time to be 10 minutes.

### Participants

Although 237 choir directors started the survey, only 125 completed the survey and only those responses were analyzed (see Table 2). Years of teaching experience ranged from 0 to 40 (M = 12.18, SD = 10.67). Ages ranged from 18 to 73 years (M = 40.16, SD = 14.10). The institutional review board at the University of Memphis exempted the study from review as it is not considered human subjects research.

**Table 2:** Demographic characteristics of participants (N = 125).

Characteristics		*n*	%
**Sex**			
Female		77	61.6
Male		48	38.4
**Teaching category**	**Years Experience**		
Preservice	0 years	13	10.4
Novice	1–6 years	38	30.4
Intermediate	7–17 years	39	31.2
Experienced	18+	35	28
**Ethnicity**			
Asian		2	1.6
Black		12	9.6
Hispanic/Latinx		2	1.6
Multirace		5	4
White		98	78
Prefer not to say		6	4.8

Next, participants were asked to listen to eight anonymous audio excerpts of high-quality choral music performances. We instructed participants to use the highest quality equipment available to them. We obtained all audio samples from public videos found on YouTube. We downloaded and converted videos to mp3 format and edited the length of each audio sample to approximately 20 to 50 seconds. For each audio sample, participants were asked to answer five Likert-scale items and one open-response item. Likert-scale items provided a number scale of 1 (Strongly Disagree) to 5 (Strongly Agree) (see Table 3). The open-response item for each audio sample asked participants to describe the choral tone and to provide information regarding what, if anything, they would do to improve the tone.

**Table 3:** Likert scale items from survey.

**This is healthy choral tone**	1 = Strongly disagree
**The choral tone is pushed/strained**	2 = Disagree
**The choral tone is bright and/or forward**	3 = Neither agree nor disagree
**This tone is dark/covered**	4 = Agree
**This is an appropriate choral tone for this style of music**	5 = Strongly agree

Audio samples contrasted in musical style, choral tone qualities, and cultural derivation (see Table 4). We selected a variety of samples we believed to be representative of choral tone familiar, proximate, and exotic to our desired participants. Recordings were of reputable community, high school, collegiate, and professional choral ensembles, but we did not disclose names of ensembles to survey participants. Our intent when selecting audio samples was for each audio sample to potentially be perceived as a model of “good” choral tone in its particular style. Additionally, the quality of the recording and other elements of performance were intended to be “good” in order to avoid distracting the listener from attention to choral tone. For more information regarding participants, the survey instrument, and audio sample selection, see [reference removed].

**Table 4:** Audio samples.

Audio Sample	Title	Composer/Arranger	Performing Ensemble
1	Sweetheart of the Sun	Eric William Barnum	Choral Arts Pure Sound
2	Dwijavanthi	Ethan Sperry	Martin High School Chamber Singers
3	Gather at the River	Stacey Gibbs	Oakwood University Aeolians
4	Sicut Cervus	Giovanni Pierluigi da Palestrina	Cambridge Singers
5	The Lioness Hunt	Lebohang “Lebo M” Morake	Mzansi Youth Choir
6	Kaval Sviri	Petar Lyondev	Bulgarian State Radio and Television Female Vocal Choir
7	Shenandoah	Robert Shaw	Robert Shaw Chorale
8	What if God	Christopher Brinson	University of Arkansas Inspirational Chorale

### Validity/Reliability

A content validity panel clarified some wording within survey items. The panel agreed the selected audio samples were appropriate for the survey. We conducted a field test. Participants (N = 4) further clarified some wording within survey items. We made revisions, addressing all suggestions provided by the content validity panel and field test participants. We used the test-retest method in the pilot test (N = 8) to measure survey reliability. We removed and/or replaced some audio samples due to low reliability and/or discovery of better quality recordings [reference removed].

### Linguistic Inquiry and Word Count

LIWC (Linguistic Inquiry and Word Count) is a computational word-counting program that connects psychological constructs with grammatical categories in language [57]. Developed by psychologist James Pennebaker, the original intent of LIWC was to model and assess psychological well-being, but the applications for this tool are much broader. While simple in design, it provides powerful categorical information about spoken and written language. LIWC generates a total word count for the document or item, and queries a static dictionary for each word in the entry to generate a proportion for that category. Its most powerful applications are to the class of lexical items called function words such as prepositions, pronouns, articles, and certain kinds of verbs. It also generates values for the general sentiment of the text or document with the categories of positive and negative emotion words, as well as anxiety and anger. LIWC has been used to assess features such as depression, deception detection, college readiness, status or hierarchy in a group, and the language of leadership. Rates of function word usage are not random, but rather are used systematically and are appropriate for modeling changes in language over time, or classifying language into typologies.

LIWC is a useful tool that can give us more nuanced insight into the ways that teachers describe choral performances, contextualized by their experience and perspective. It provides insight about *how* respondents are talking about the musical selections. It also provides a robustness check for the categories of interest, like experience and age, that we expect will differentiate between teachers’ evaluations of the musical selections. Topic modeling, on the other hand, provides information about *what* respondents are saying. Used together, these methods can help to quantify the imprecise lexical categories that represent the gap between theory and practice in judging the quality (in terms of health and appropriateness) of musical selections across a range of genres. Because judging choral tone is so subjective, these methods provide an empirical basis for how practitioners define this concept.

For this study we selected the following LIWC variables to include in our empirical model: word count; auxiliary verbs (“helping” verbs); negations (not, nor); adjectives (beautiful, small); comparisons (better, worse); positive and negative emotion words; and discrepancies (should, ought). In Table 5 we provide a summary of the language variables used in our empirical models.

**Table 5:** Summary of language variables.

Variable	Obs	Mean	Std. Dev.	Min	Max
Word count	1000	22.49	20.02	0.00	146.00
Auxiliary verb	1000	7.11	6.27	0.00	37.50
Negate	1000	2.51	8.69	0.00	100.00
Adjective	1000	10.72	14.12	0.00	100.00
Compare	1000	3.25	5.45	0.00	50.00
Positive emotion	1000	8.20	13.97	0.00	100.00
Negative emotion	1000	1.14	3.31	0.00	50.00
Discrepancy	1000	2.12	3.29	0.00	20.00
Topic 1	1000	7.96	1.48	3.65	15.72
Topic 2	1000	16.54	2.46	9.48	30.97
Topic 3	1000	7.50	1.46	3.44	18.53
Topic 4	1000	16.12	2.15	8.44	24.39
Topic 5	1000	5.73	1.34	3.10	16.69
Topic 6	1000	5.14	1.35	2.77	19.55
Topic 7	1000	6.95	1.66	3.33	18.75
Topic 8	1000	5.00	1.33	2.39	13.44
Topic 9	1000	4.25	1.41	2.03	25.21
Topic 10	1000	24.82	2.66	14.41	37.55

### Topic modeling

Topic modeling is a way to derive categories or themes of topics from a corpus, or set of documents [58]. For this study, the open ended survey responses (n = 1000) serves as our corpus; we had 125 participants who each completed open-ended responses for the eight different audio samples. Also called Latent Dirichlet Allocation (LDA), topic modeling provides a probabilistic model of words across a number of topics; for this study we specified ten topics. Each word in the corpus has some probability of being in each topic; words that tend to co-occur have a higher probability of being in the same topic. Words are not sorted into topics based on their semantic similarity, although semantically related words may appear in the same topic due to their context within the documents in the corpus. As Nelson (2017) notes, topic modeling is a form of computational grounded theory; the LDA algorithm used by the computer efficiently sorts the terms into a specified number of topics, and the researchers qualitatively interpret the meaning of the key terms in the topic [59]. Table 6 (below) lists twenty key terms for ten topics derived from the open-ended survey responses. The probability distribution of topics across documents can be used in empirical models to demonstrate their relationship to a quantity of interest, such as Likert scale items. In doing this, we can answer questions about *how* survey respondents described musical selections that they rated as healthy or appropriate.

**Table 6:** Topic key terms for open-ended responses.

Topic	Top Words
Mechanism	singing voice sung great range tones recording performance *tension nasal* pitch piece encourage *supported* genre *soft free* mechanism sing perfect
Gospel	style music sounds healthy choral piece vocal gospel production sing don sound unhealthy *pushed* strained good tone change *pushing* ensemble
Mature	sound choir make throat *relax* sopranos mature line renaissance supported *full* air classical men sections chest tone sample time excellent
Stylistically appropriate	*bright* sound forward *nasal* pushed forced *warm* song stylistically wouldn larynx supported tone *mask* support correct hear parts open *volume*
American tone	placement *clear* back nice focus dynamics round feel vibrato *louder* men *heavy american traditional* unified focused color long makes tones
Culture	singing *culture* brightness improve feel blend type sounds practice forward richness vocal mouth fine unison *lowered asian* pieces kind text
Choral blend and balance	voices strained *upper* notes *lower* vibrato register *blend treble balance soprano* men strong love improve loud lot sing natural rounded
Men	beautiful placement sounded understand times song *bass* balanced consonants men darker dynamic pronunciation feels back pushed accurate end individual isn
Range	tenors sopranos basses group opinion musical doesn side low piece ranges personally completely *chest* strident tune spiritual brighten *free* vibrato
Resonance	tone bit singers dark *forward* work vowels covered straight voice slightly breath full open *space* sound quality resonance darker *rich*

Table 7 provides the list of terms generated by choral directors and practitioners in their open-ended survey responses to describe tone in choral performances across a range of genres, and Table 6 provides the list of terms identified by McKinney (2005) for good and poor tone [11]. While there is some overlap between “textbook” terminology and the descriptions provided by choral directors, there are clear distinctions between them. In the practitioner-defined terms, we see the added categories of resonators (nasal, mask, chest), tension (pushed, relax), richness (full, warm), clarity (clear), weight (heavy), position (upper, lower), choral purity (blend, balance), geography (culture, American, Asian, traditional), and register (soprano, bass, treble). Practitioners from our survey are tapping into a much broader range of descriptive categories than are provided in the literature, signaling a gap between theory and practice.

**Table 7:** Good and poor tone (McKinney, 2005 [11]).

**Good tone**	freely produced; pleasant to listen to; loud enough to be heard easily; rich, ringing, and resonant; energy flows smoothly from note to note; consistently produced; vibrant, dynamic, and alive; flexibly expressive
**Poor tone**	constricted, forced, or strained; strident or rasping; too loud, resembling shouting or yelling; hoarse; breathy; weak, colorless, or devitalized; inconsistently produced; shaky or wobbly

To better understand the relationship between choral director experience and culturally diverse choral performances, we analyze the survey respondents’ ratings in the context of the language they use to describe performances. Choral directors were asked to decide whether choral tone in a particular performance was healthy and appropriate by choosing one of the following responses: strongly disagree (1); somewhat disagree (2); neither agree nor disagree (3); somewhat agree (4); or strongly agree (5). We include a Kruskal-Wallis one-way analysis of variance test in the Supporting Information to ascertain differences in responses of each rating by years of teaching experience. We use ordinal logistic regression and logistic regressions for linguistic and topical features. The full regression tables are provided in the Supporting Information for the marginal effects graphs we provide below.

## Results

Ratings for both health and appropriateness (Hypotheses 1a and 1b) generally follow the same trends. Based on the marginal effects, teachers with less classroom experience are more likely to rate the performances as healthy or appropriate. At each increasing level of experience, teachers are less likely to rate the performances as healthy and appropriate.

### Ordinal logistic regression: Health and appropriateness

**Fig 1:**
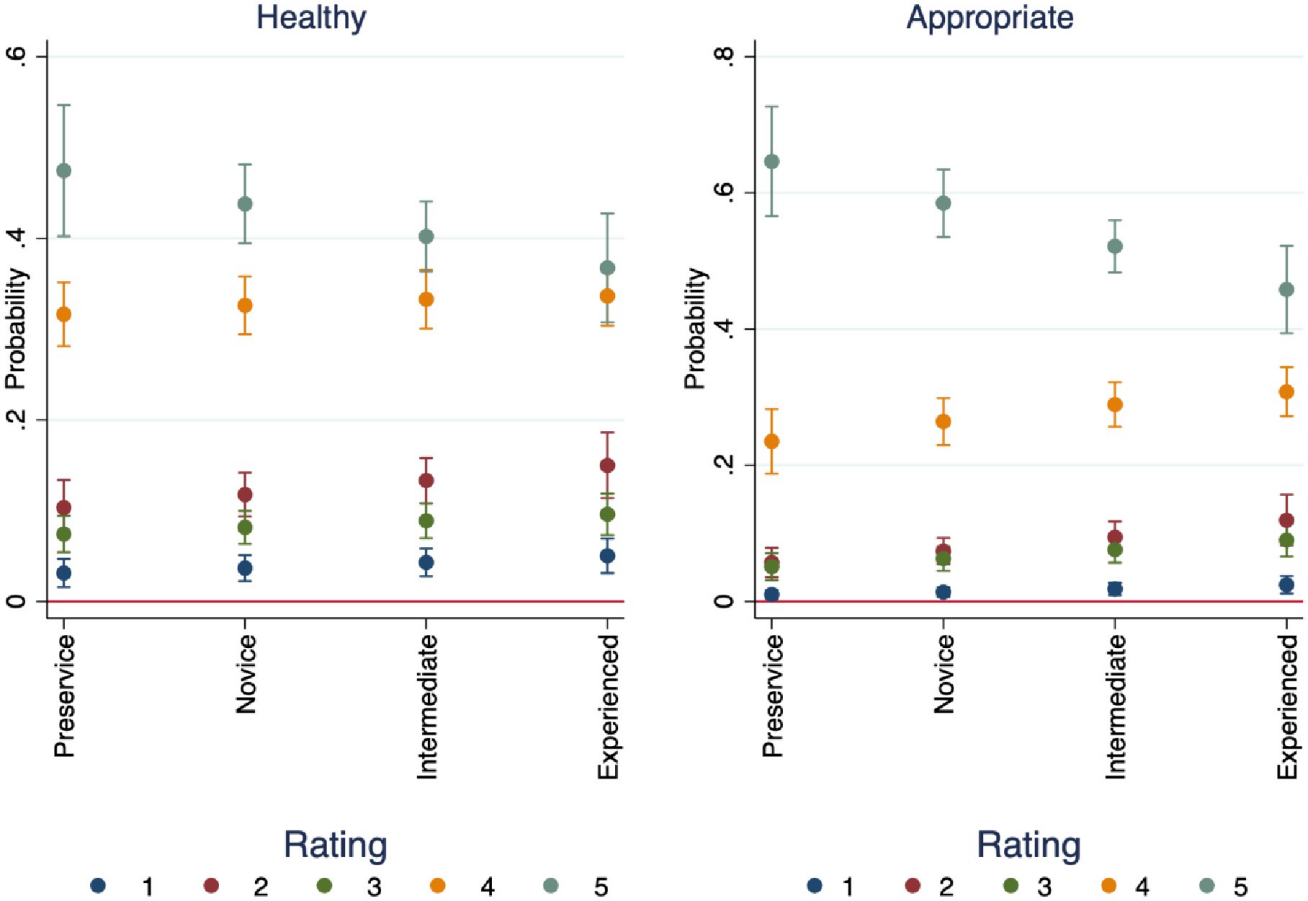
Marginal effects of years’ experience on probability of rating audio samples as healthy and appropriate.

We performed an ordinal logistic regression where the dependent variable is an ordinal ranking of health (1 meaning least healthy and 5 meaning most healthy) and appropriateness (where 1 is least appropriate and 5 is most appropriate). Fig 1 plots the marginal effects of the respondents’ teaching experience on the probability of rating all audio samples overall as healthy or unhealthy. Teachers with fewer years of experience tend to rate the performances as healthier and more appropriate overall. Furthermore, there is a linear trend across experience levels and assigning the highest score for health and appropriateness, where teachers with more experience rate performances lower sequentially. Interestingly however, the second-highest rating (4) shows the opposite trend, showing a linear increase across experience levels. There is also more heterogeneity of ratings for teachers with the least experience, with more variation among in assigning ratings of health.

**Fig 2:**
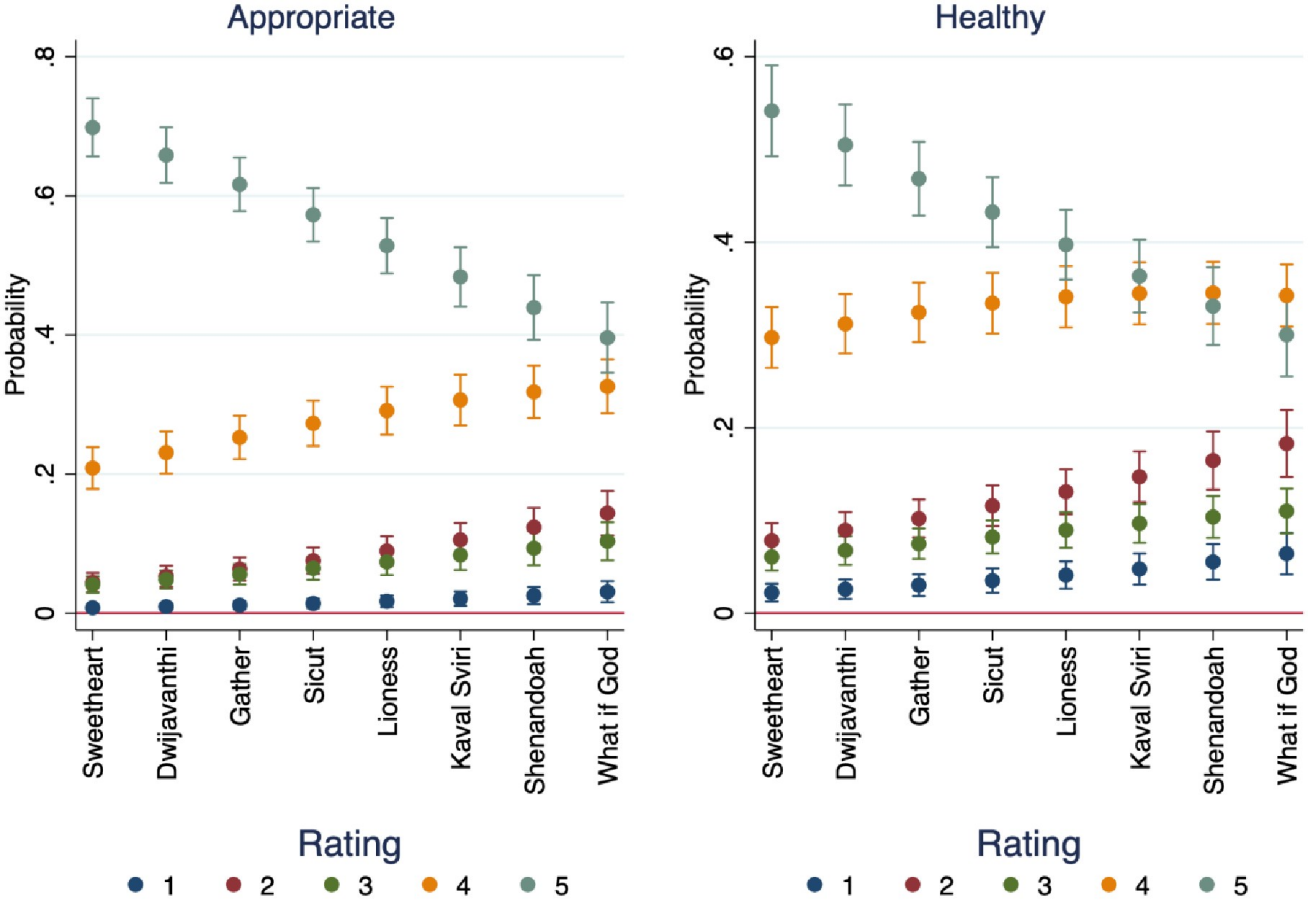
Probability of rating audio samples as healthy and appropriate, by audio sample.

Fig 2 shows the probability of rating audio samples as healthy and appropriate overall. There are clear trends between overall ratings of the audio samples, which may be evidence of the linear presentation of the audio samples. The order of audio samples was presented consistently across subjects, not randomized. However, the audio samples themselves were presented in random order, not grouped by category. Participants were more likely to rate earlier audio samples higher than later ones, and later audio samples lower than earlier ones. Most importantly, we find statistically significant differences across quantities of interest like the experience divides, even given this apparent linear dependence.

Only audio samples one (Sweetheart of the Sun) and four (Sicut Cervus) had median scores of five, indicating strongest agreement, that the performances were healthy and appropriate. Both of these audio samples were from the most familiar, Western presentation style of choral performance, which lends support to our hypotheses that respondents will rate the tone of more culturally proximate styles higher than those most different from their experience and training.

**Fig 3:**
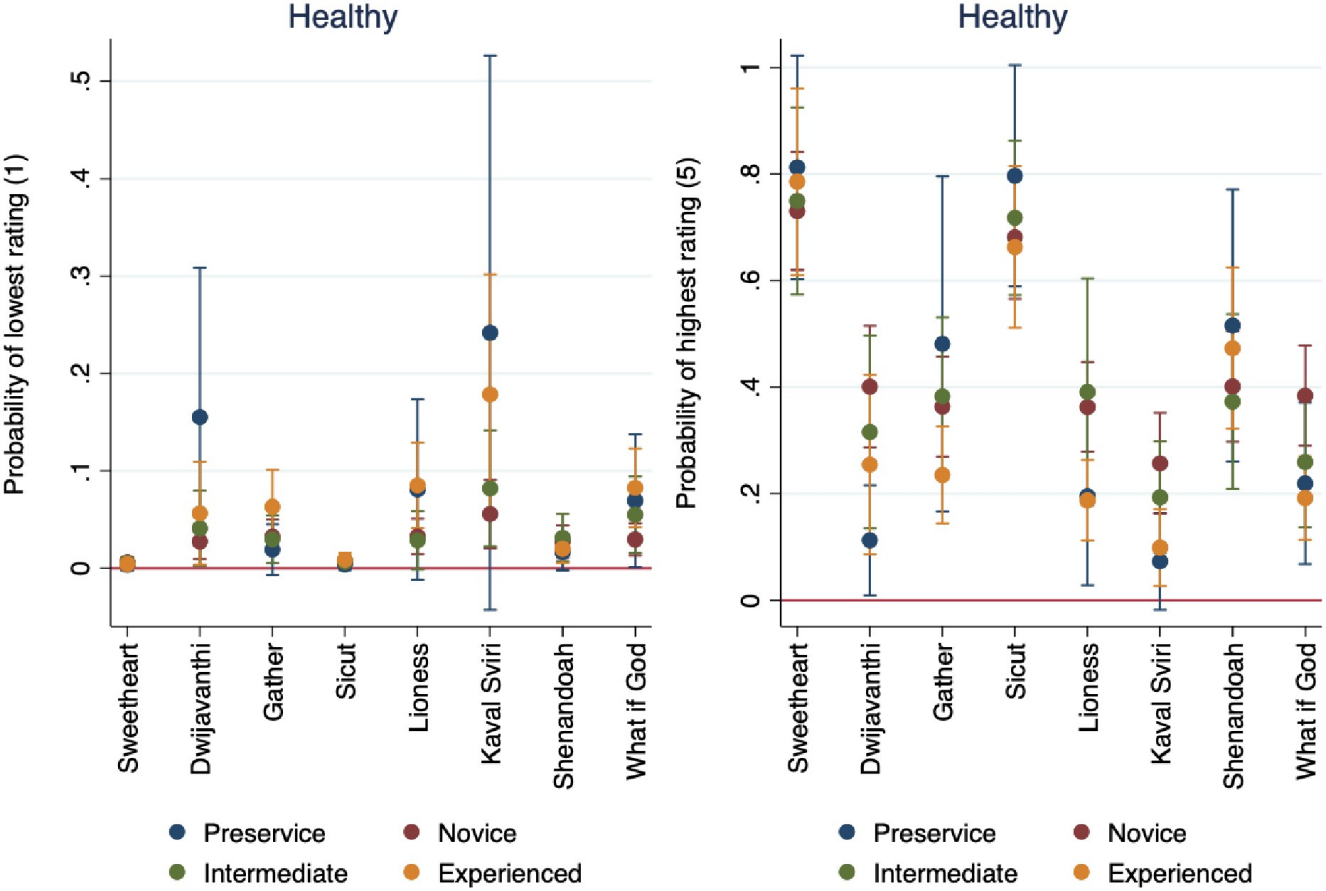
Marginal effects of years’ experience on probability of rating audio samples as healthy, by audio sample.

**Fig 4:**
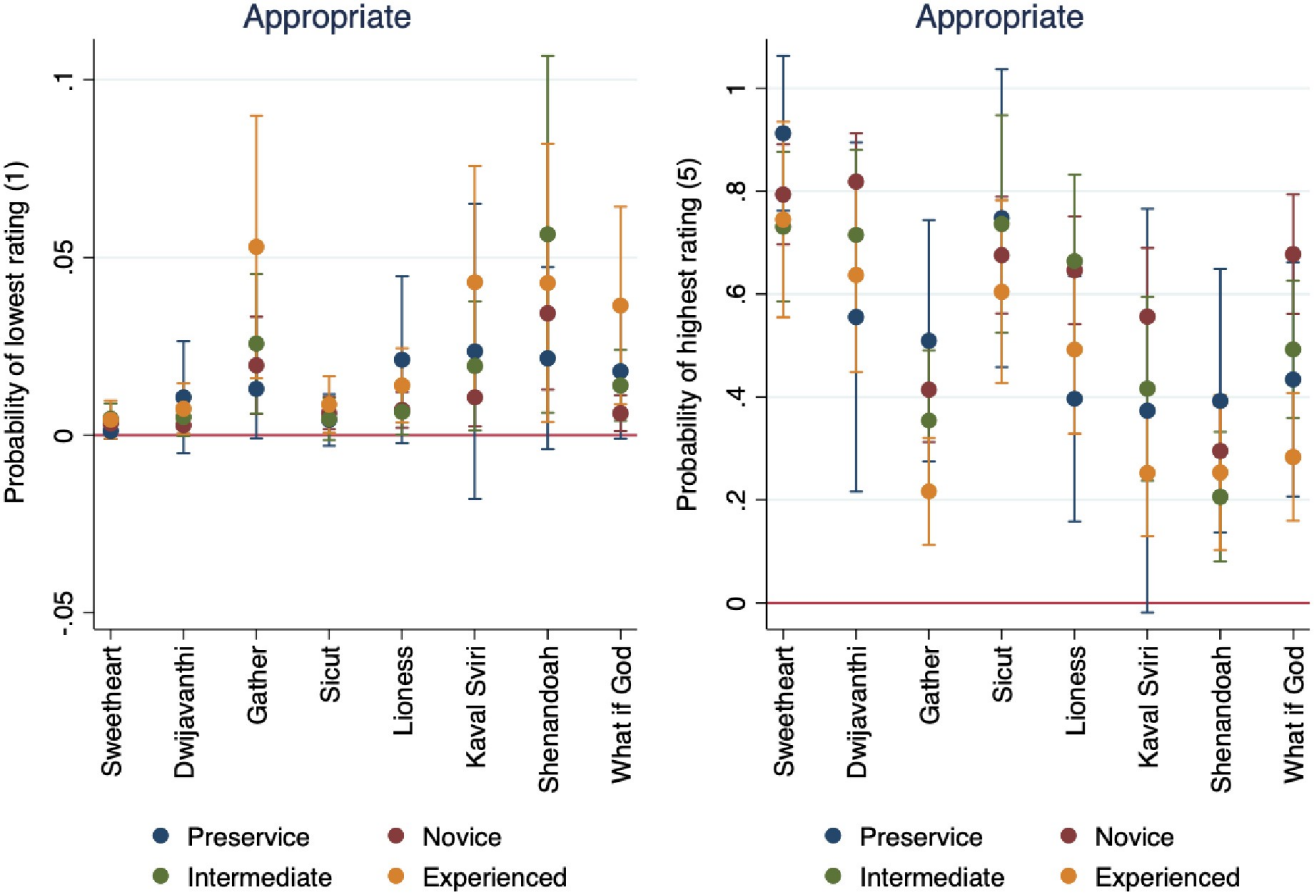
Marginal effects of years’ experience on probability of rating audio samples as appropriate, by audio sample.

Figs 3 and 4 show the effects of years’ teaching experience on the probability of rating audio samples as healthy and appropriate, across the audio samples presented. In audio samples two, five, six, and eight, we see that the overall ratings for health were lower than the overall ratings for appropriateness. The second audio sample is “Dwijavanthi” from India, and the fifth audio sample is “The Lioness Hunt” from South Africa. The sixth audio sample is “Kaval Sviri” from Bulgaria, and the eighth audio sample is the American Black Gospel song “What if God.” These four selections were chosen as samples because of their difference from traditional Western choral art pieces. There is similar pattern across these four audio samples, namely that respondents tend to rate them as less healthy, but more appropriate. This provides partial support for our hypotheses that respondents would rate choral tone in culturally familiar performances as healthier and more appropriate than choral tone in styles different from their predicted experience and training.

**Fig 5:**
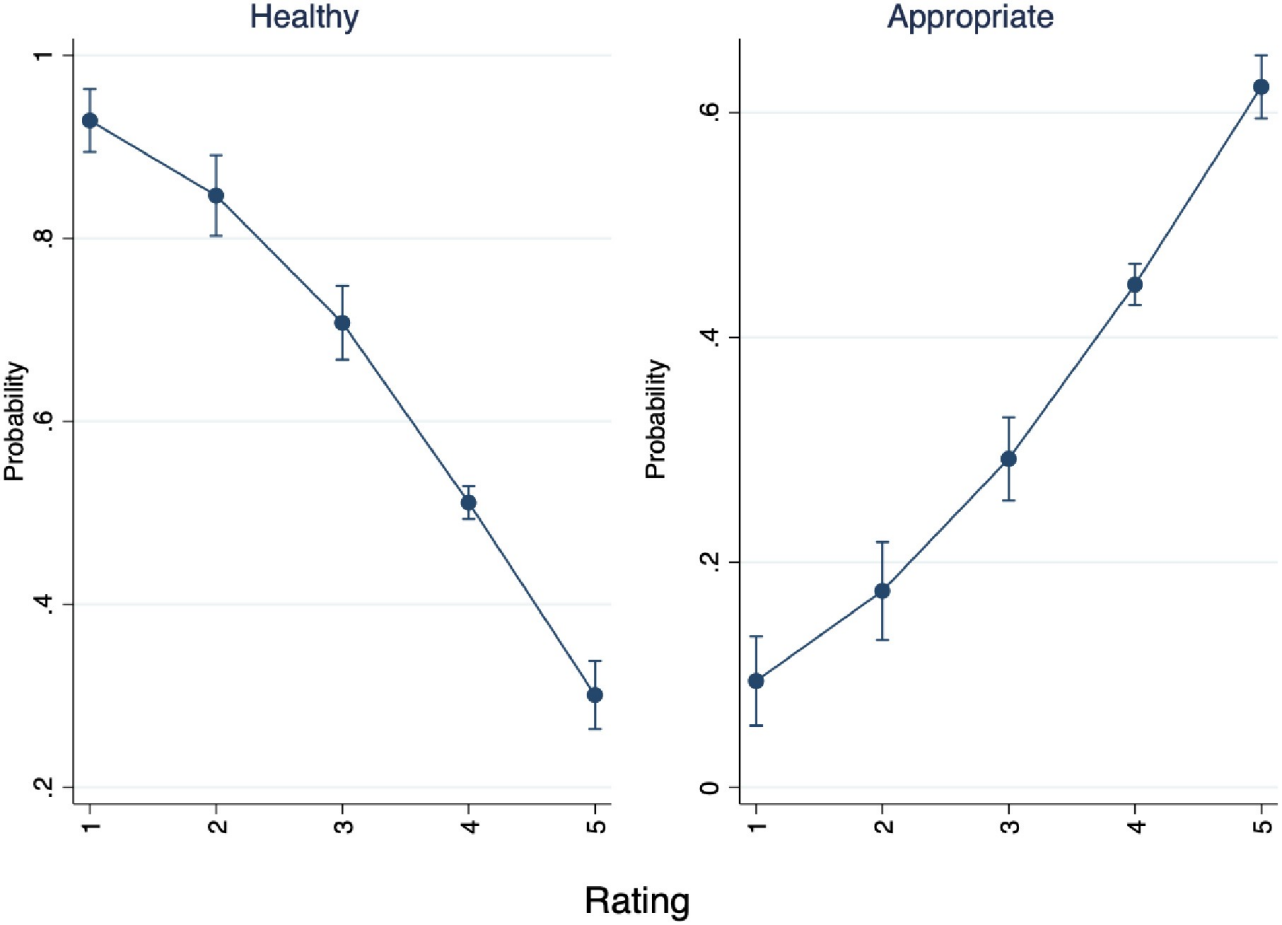
Marginal effects of Western and non-Western audio selections for health and appropriateness.

In Fig 5 we see the graphed marginal effects of a logistic regression where the dependent variable is dichotomous, capturing whether the audio selection is from a traditionally Western or non-Western tradition (Hypothesis 2). This analysis provides some generalizability for our findings to the aggregate categories of Western and non-Western genres, rather than relying on specific musical selections. Audio samples 2, 5, 6, and 8 were coded as “1, non-Western” and 1, 3, 4, and 7 as “0, Western”.

In Fig 5, we see that all respondents were more likely to rate non-Western selections as less healthy, following an inverse linear trend. On the other hand, we see that all respondents were more likely to rate non-Western selections as more appropriate. The probability of a musical selection being rated as non-Western is about 30% if the respondent gave the value of 5. Conversely, if a respondent rated a musical selection with the lowest rating, 1, the piece has nearly a 90% chance of being rated as non-Western. The implications of these findings are that lower-rated pieces are more likely to be from non-Western genres, and less likely to be perceived as healthy than their Western counterparts. Fig 5 also shows the inverse relationship with appropriateness ratings. The probability of a musical selection being rated as non-Western is about 10% if a respondent gives the piece the lowest rating, 1. On the other hand, if the piece is assigned the highest value of 5, it has about a 60% chance of being classified as non-Western. The generalizable findings are that non-Western selections are less likely to be rated as healthy, but more likely to be rated as appropriate.

**Fig 6:**
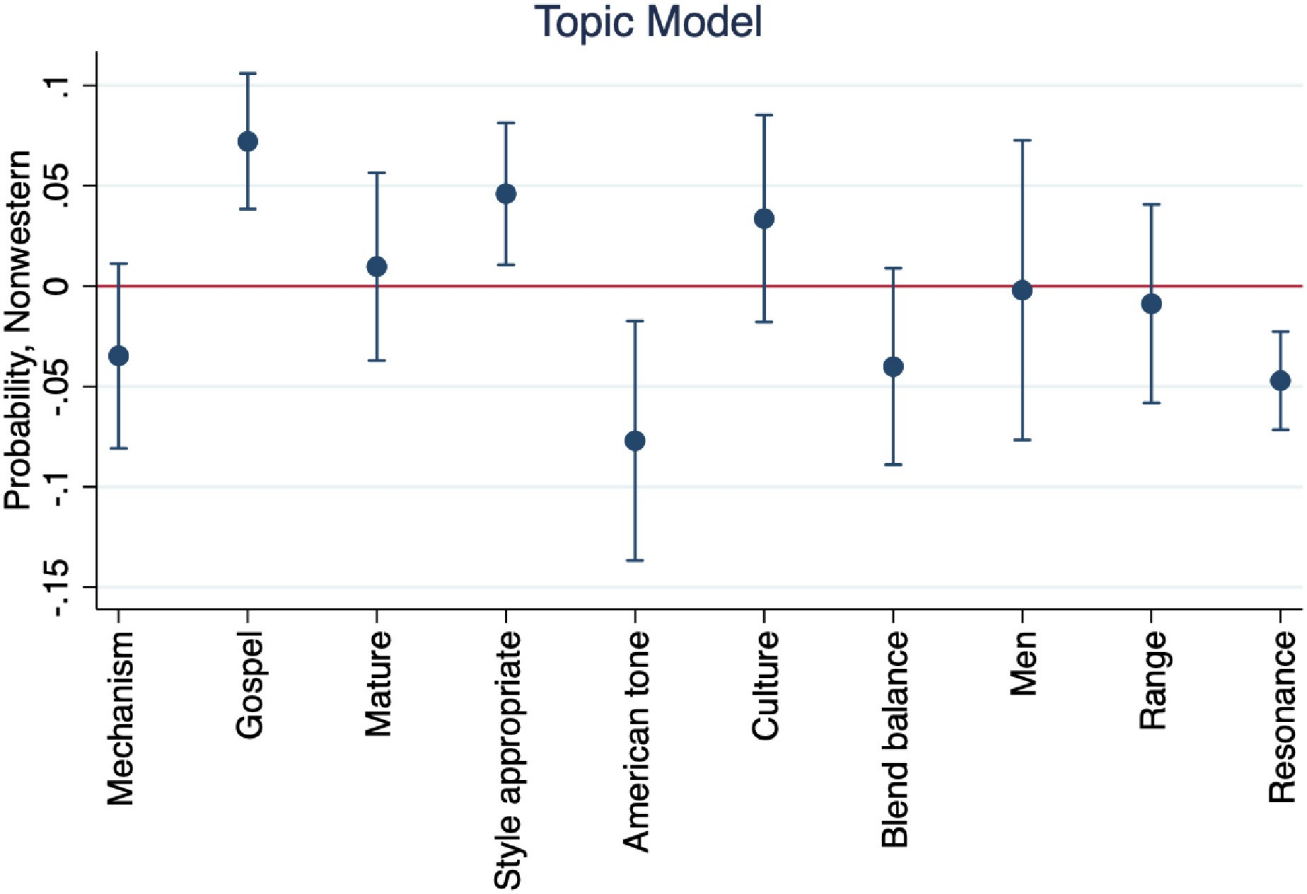
Marginal effects of topics on the probability of non-Western music.

In Fig 6 we see the marginal effects of the ten topics in our model on the probability of musical selections being of non-Western origin (Hypothesis 2). We see that Topics 2 and 4, *Mechanism* and *Mature* are statistically significant predictors of non-Western songs, while *American Tone* and *Resonance* are statistically significantly less likely to be used to describe non-Western music. Words in *Mechanism* and *Mature* topics relate to the anatomy and desired result of tone production. We interpret its significance in regard to non-Western performances to indicate an encouragement toward healthy tone production. Naturally, *American Tone* was more significant in describing Western music, as the terms all centered around characteristically Western qualities. *Resonance* may be more significant Western performance descriptions due to the Western prioritization of resonance and how it can be achieved.

**Fig 7:**
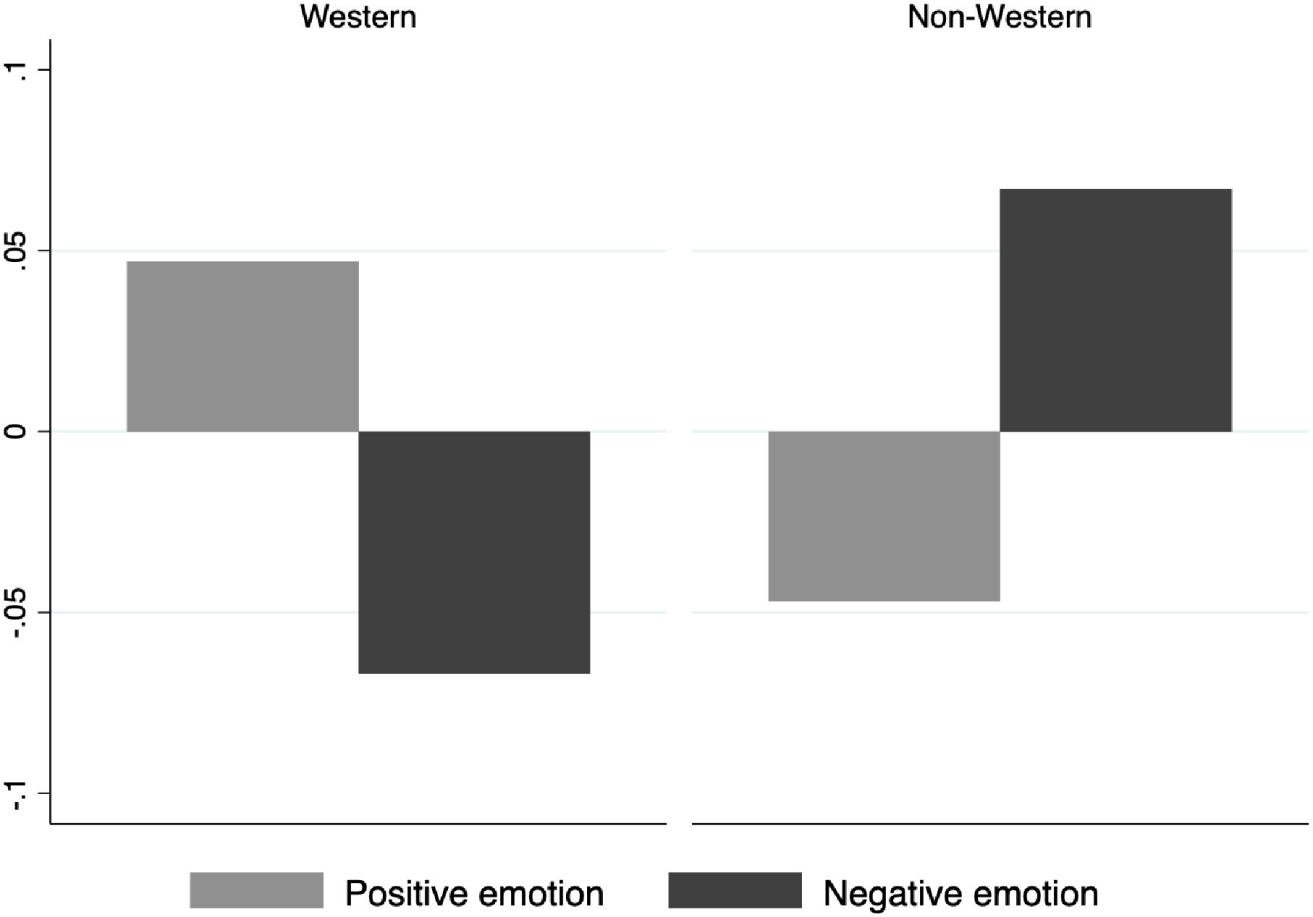
Positive and negative emotion language use by respondents across cultural categories.

In Fig 7 we see the difference between use of positive and negative emotion to describe choral tone in traditional Western and non-Western musical selections (Hypothesis 3). All respondents used more positive emotion and less negative emotion to describe the tone in traditional Western selections, and less positive and more negative emotion to describe the tone in non-Western selections.

**Fig 8:**
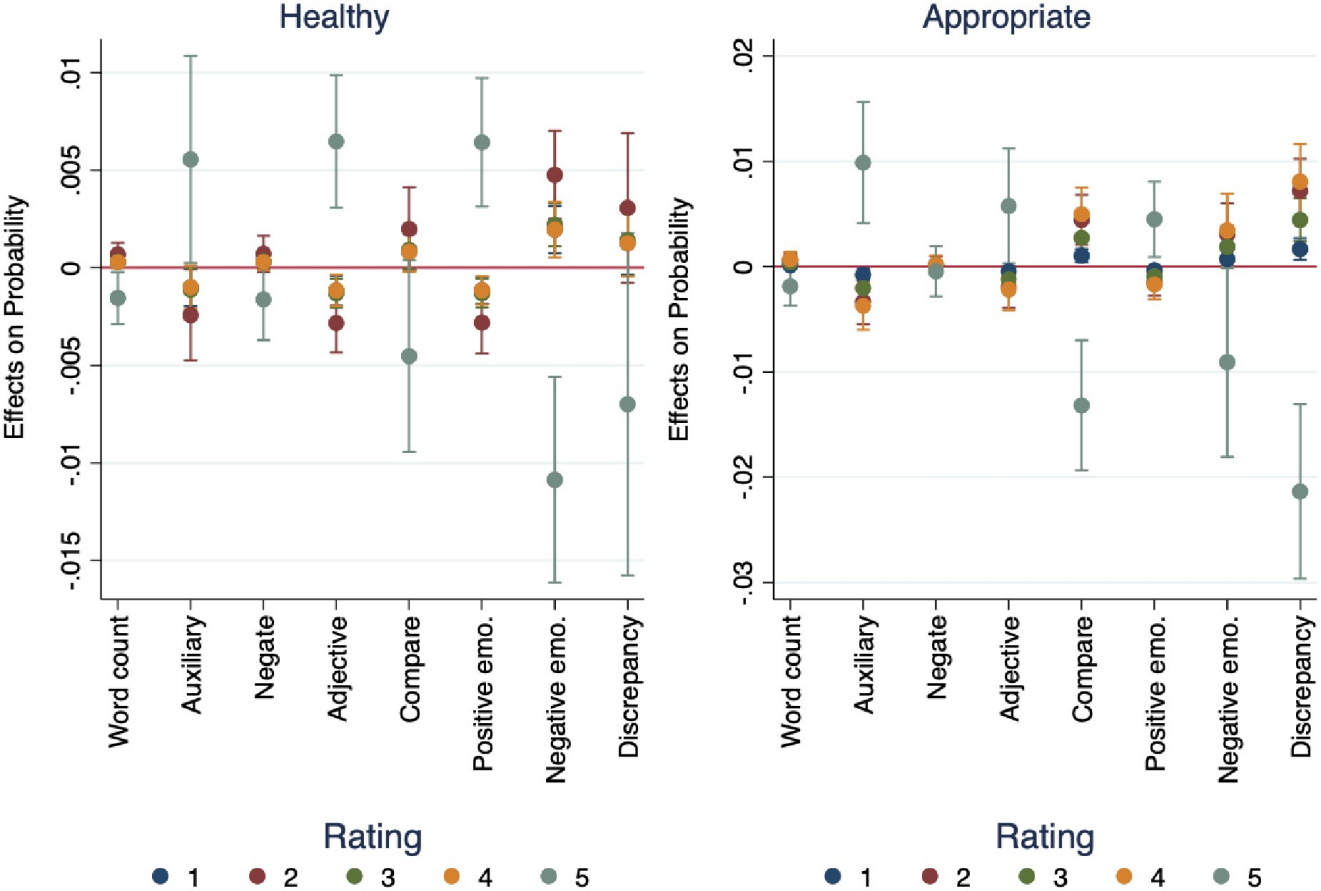
Marginal effects language use on probability of rating audio samples as healthy and appropriate.

In Fig 8, we see similar patterns in how choral teachers describe the health and appropriateness of the choral tone in performances they listened to (Hypotheses 1a, 1b, and 3). For the “strongly agree” choice (meaning, the performances were considered healthy and appropriate), the respondents used fewer words overall. We interpret this to mean that for performances they deemed healthy and appropriate, the respondents had less to say. For respondents who rated the performances (strongly agree) as healthy and appropriate, they tended to use fewer words overall, fewer negations, fewer comparisons, and less negative emotion. They also tended to use more auxiliary (“helping”) verbs, more adjectives, and more positive emotion in their open-ended responses for each audio sample item. These findings align with our theoretical hypotheses that lower status, newer teachers would use more words, and more descriptions, than higher status, more experienced teachers [56].

**Fig 9:**
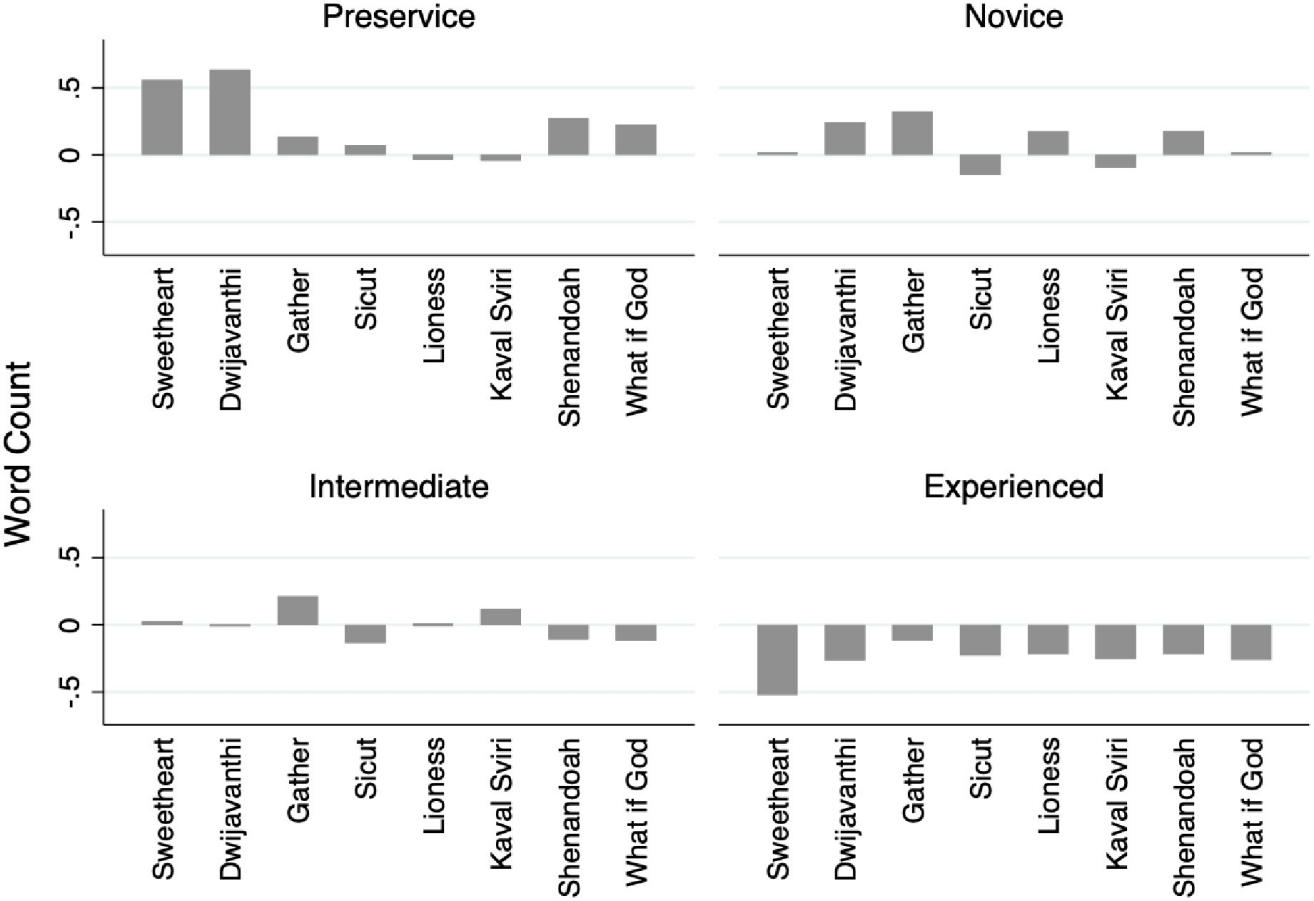
Mean word count by experience level and musical selection.

In Fig 9 we see evidence for seniority, operationalized as years’ teaching experience (Hypothesis 1a, 1b, and 3). Research has established that people with lower status or less authority tend to write or speak more words than do those in positions of higher status or authority [57]. We see evidence of this from the open-ended writing samples from survey respondents, where pre-service teachers tend to use the most words in their open-ended survey responses, and teachers with the most experience (more than eighteen years) tend to write the fewest across audio samples. This provides evidence that novice and experienced teachers have different perspectives on analyzing musical performances.

Table 8 provides an overview of support for our hypotheses.

**Table 8:** Summary of expectations and findings.

Hypothesis	Supported	Description
**Experience**	1a: Unsupported 1b: Supported	Experienced choral directors rated performances lower in health and appropriateness than those with less experience.
**Culture**	2: Partially supported	Choral directors rated culturally familiar performances as healthier, but not more appropriate, than those different from their experience and training.
**Language**	3a: Supported 3b: Supported 3c: Supported	Choral directors used more descriptors, more positive emotion, and less negative emotion for performances they found healthier and more appropriate. Choral directors used fewer discrepancy terms for performances they found healthier and more appropriate. Choral directors with more experience used fewer words than those with less experience.

## Discussion

The purpose of this study was to identify what factors influence choral directors’ descriptions of choral tone in order to enable choir directors to mindfully approach teaching healthy and stylistically appropriate tone production. From our survey results we have learned that experience is a stronger predictor of choral directors’ evaluations of choral tone across a range of genres than is their cultural background. Perception of multicultural music, it seems, is more a function of experience than of exposure. In other words, we observe differences along the experiential divides. We do not interpret this to mean that experienced choral directors are incapable of appropriately teaching non-Western tone production techniques. On the contrary, we suggest that having less vague terminology will enable teachers from all experience levels to better evaluate non-Western music. Similarly, with increased exposure in teacher preparation programs to courses in ethnomusicology, choral literature, and vocal pedagogy, teachers will gain more practical experience in evaluating choral tone within a wide variety of music. From the open-ended survey responses we also learned that language is a predictor of status and experience (word count), and that choir teachers have a much greater vocabulary repertoire and more thematic categories in classifying and describing choral performance than is found in the canonical literature [11–13,31,41].

In scholastic choral music, there are no objective standards for tone and there is no fixed lexicon for describing good and poor musical performances. Governing agencies are mandating that multicultural music education be taught and performed, but have not yet created a sustainable structure to enable authenticity in multicultural music performance at scholastic levels. In order to meet accreditation standards, university schools of music must provide education in music from a variety of cultures, but the accreditation standards do not specify *how*. Further, teachers are given conflicting instructions: they are to engage in learning multicultural music, but when they describe vocal adjustments, not to draw on their own personal experience [12,31]. When preservice teachers graduate and begin teaching middle or high school choir, they are expected to abide by national standards. Standards for middle and high school choir students are similar to university accreditation standards in that they are vague to allow teachers the liberty and flexibility to most appropriately serve their students. Yet this lack of specificity can enable unprepared teachers to lead students into inappropriate performance practice, particularly in tone production, which varies from culture to culture.

Choir directors independently—and within their own communities of practice—develop theories of healthy and appropriate tone production as they learn from their choir directors and voice instructors. From their experiences, they teach their choir students how to produce what they consider to be healthy and appropriate tone quality. We treat choir directors’ assessments of their, and other, ensembles as objective, but in reality they are strongly subjective with little oversight or recourse or remedy due to strong variation in training and lack of clearly defined language to describe tone. Underprepared choir directors may inadvertently model higher value of culturally-familiar music over multicultural music to their students, exacerbating musical ethnocentrism, and thereby, bias. As student populations become increasingly more diverse, the need for culturally responsive, and thereby multicultural, music education in teacher preparation programs escalates.

## Conclusions

The implications for this study are pedagogically important: contemporary music classes in the United States are increasingly comprised of students from diverse backgrounds. This includes not only race and ethnicity, but also country of origin, and primary language. Wong et al., estimated cultural differences in music perception of speakers of tonal and non-tonal languages, finding that speakers of tonal languages do better at processing musical pitch [10]. The standards of tonal beauty as taught in Western curriculum may create opportunities for ethnocentrism and bias, whether intentionally or implicitly, and this may harm students. By analyzing how teachers perceive choral tone across a variety of styles, we can begin to understand what features of teacher training can better prepare choir teachers and directors for their diverse classrooms and best serve students from many backgrounds.

One solution is to better prepare choral music educators to teach multicultural music and relevant practices. This can be accomplished through post-licensure trainings, professional development, and continuing education units (CEU) required to maintain a teaching license. These trainings should include musicians and practitioners fluent in the target style of music who can best describe and convey the standards of health and appropriateness in that culture. Fluency, or at least proficiency, in a variety of musical cultures should also help mitigate against the hazards of providing inaccurate information or guidance to students. Preservice choir directors should not only learn about appropriate tone practices in a variety of styles from a variety of cultures, they should learn effective ways to convey those methods to their future students. With a more precise lexical “toolbox” and a more thorough education in multicultural tone practices, novice choir directors will be more prepared to appropriately educate their students from day one in the classroom.

There are many benefits to implementing an institutional response to address issues with multicultural music and its practices. From a pedagogical perspective, there are benefits to clarifying and defining the issue landscape, defining the terminology for describing healthy and appropriate choral tone. Teachers with experience can benefit from continuing education in tone terminology and appropriate tone production practices of a variety of cultures. Professional development can help raise the cultural competence of choral music educators if it is structured in such a way as to be comprehensively uniform. Additionally, standards can remain flexible and simply be used to design curricula that meet the needs of choral music programs and the students that populate them.

Students will also benefit from increased cultural awareness, empathy, openness to new experiences, and the authenticity of being accurately trained in a variety of styles. Broadly speaking, the profession will benefit from increased diversity of choral programming, with less trepidation about receiving negative feedback or judgment from professional adjudicators. Increased proficiency in multicultural musical performance will empower the next generation of choral directors to attempt new music, through an institutional framework that supports the practice of teaching a broader range of musical styles.

## Supporting information

S1 Table Kruskal-Wallis one-way ANOVA results. (DOCX)

S2 Table Base model regression results for Figs 1–5, 7 and 8. (DOCX)

S3 Table Topic model regression results for Fig 6. (DOCX)

S4 Table Logistic regression for nonwestern choral selections. (DOCX)
