# Supplementary material for: Effects of teaching experience and culture on choral directors’ descriptions of choral tone
Source: PLoS One. 2021 Dec 7;16(12):e0256587. doi: 10.1371/journal.pone.0256587 (PMC8651130; doi:10.1371/journal.pone.0256587)
Supplement: S1 Table — (DOCX) [file pone.0256587.s001.docx]

*S1 Table. Kruskal-Wallis one-way ANOVA results*

| Audio Sample | Results |
| --- | --- |
| 1 | *H* = 1.16, 3 *df*, *p* = .764, *η*^2^ = -0.02 |
| 2 | *H* = 1.89, 3 *df*, *p* = .597, *η*^2^ = -0.01 |
| 3*   Experienced (*Mdn* = 3.32), Preservice (*Mdn* = 4.11)   Experienced (*Mdn* = 3.32), Novice (*Mdn* = 3.95)   Experienced (*Mdn* = 3.32), Intermediate (*Mdn* = 4.26) | *H* = 14.05, 3 *df*, *p* = .003, *η*^2^ = 0.09  *H* = 25.27, 3 *df*, *p* = .022, *η*^2^ = 0.18  *H* = 17.14, 3 *df*, *p* = .032, *η*^2^ = 0.12  *H* = 28.63, 3 *df*, *p* = <.001, *η*^2^ = 0.21 |
| 4 | *H* = 3.58, 3 *df*, *p* = .311, *η*^2^ = 0.00 |
| 5 | *H* = 4.61, 3 *df*, *p* = .202, *η*^2^ = 0.01 |
| 6 | *H* = 7.71, 3 *df*, *p* = .052, *η*^2^ = 0.04 |
| 7 | *H* = .20, 3 *df*, *p* = .977, *η*^2^ = -0.02 |
| 8*   Experienced (*Mdn* = 4.04), Novice (*Mdn* = 4.78)   Experienced (*Mdn* = 4.04), Intermediate (*Mdn* = 4.45)   Novice (*Mdn* = 4.78), Preservice (*Mdn* = 4.27)   Novice (*Mdn* = 4.78), Intermediate (*Mdn* = 4.45) | *H* = 17.37, 3 *df*, *p* = .001, *η*^2^ = 0.12  *H* = 31.26, 3 *df*, *p* = <.001, *η*^2^ = 0.23  *H* = 15.49, 3 *df*, *p* = .042, *η*^2^ = 0.10  *H* = 23.63, 3 *df*, *p* = .025, *η*^2^ = 0.17  *H* = 15.77, 3 *df*, *p* = .035, *η*^2^ = 0.11 |

*findings significant at p<.05
