# Supplementary material for: Effects of teaching experience and culture on choral directors’ descriptions of choral tone
Source: PLoS One. 2021 Dec 7;16(12):e0256587. doi: 10.1371/journal.pone.0256587 (PMC8651130; doi:10.1371/journal.pone.0256587)
Supplement: S2 Table — (DOCX) [file pone.0256587.s002.docx]

*S2 Table. Base model regression results for Figures 1, 2, 3, 4, 5, 7, and 8*

|  | Healthy |  | Appropriate |  |
| --- | --- | --- | --- | --- |
|  | Coeff. | Std.Err. | Coeff. | Std.Err. |
| Word count | -.0072748* | 0.003 | -.0088787* | 0.005 |
| Auxiliary verbs | .0260064* | 0.013 | .0468308** | 0.014 |
| Negate | -0.008 | 0.005 | -0.002 | 0.006 |
| Adjective | .0303492*** | 0.008 | .0272878* | 0.014 |
| Compare | -0.021 | 0.012 | -.0625228*** | 0.016 |
| Positive emotion | .0301287*** | 0.008 | .0213303* | 0.009 |
| Negative emotion | -.0509735*** | 0.012 | -.0430386* | 0.022 |
| Discrepancy | -0.033 | 0.021 | -.101312*** | 0.020 |
| Audio sample | -.1597827*** | 0.022 | -.203093*** | 0.020 |
| Sex | 0.220 | 0.161 | 0.148 | 0.177 |
| Generation | -0.167 | 0.088 | -.2974181** | 0.104 |
| cut1 | -4.116535*** | 0.400 | -5.949306*** | 0.490 |
| cut2 | -2.50146*** | 0.340 | -3.968466*** | 0.424 |
| cut3 | -1.942499*** | 0.331 | -3.315502*** | 0.418 |
| cut4 | -0.370 | 0.317 | -1.767079*** | 0.410 |
| N. of cases | 1000.000 |  | 1000.000 |  |
| * p<0.05, ** p<0.01,*** p<0.001 | | |  |  |
