# Supplementary material for: Effects of teaching experience and culture on choral directors’ descriptions of choral tone
Source: PLoS One. 2021 Dec 7;16(12):e0256587. doi: 10.1371/journal.pone.0256587 (PMC8651130; doi:10.1371/journal.pone.0256587)
Supplement: S3 Table — (DOCX) [file pone.0256587.s003.docx]

*S3 Table. Topic model regression results for Figure 6*

|  |  | Healthy |  | Appropriate |  |
| --- | --- | --- | --- | --- | --- |
|  |  | Coeff. | Std.Err. | Coeff. | Std.Err. |
| 1 Mechanism | | -3.900 | 4.815 | -0.371 | 5.792 |
| 2 Gospel |  | -10.91377*** | 3.179 | 6.316 | 3.626 |
| 3 Mature |  | 2.635 | 4.750 | 5.169 | 5.113 |
| 4 Style appropriate | | -4.852 | 3.645 | 0.736 | 3.912 |
| 5 American tone | | -2.930 | 4.110 | -7.044 | 4.430 |
| 6 Culture |  | -12.86975** | 4.403 | 1.394 | 4.377 |
| 7 Blend balance | | -6.492 | 3.789 | -8.621916* | 3.845 |
| 8 Men |  | -4.356 | 4.735 | 2.763 | 4.682 |
| 9 Range |  | -5.411 | 4.443 | -5.348 | 4.361 |
| 10 Resonance | | 0.000 | . | 0.000 | . |
| Audio sample | | -.1698594*** | 0.022 | -.188149*** | 0.023 |
| Experience |  | -0.157 | 0.089 | -.2806524** | 0.099 |
| cut1 |  | -8.869016*** | 1.849 | -5.29864** | 1.980 |
| cut2 |  | -7.271582*** | 1.833 | -3.347 | 1.975 |
| cut3 |  | -6.728124*** | 1.834 | -2.713 | 1.974 |
| cut4 |  | -5.24236** | 1.836 | -1.269 | 1.971 |
| N. of cases |  | 1000.000 |  | 1000.000 |  |
| * p<0.05, ** p<0.01,*** p<0.001 | | |  |  |  |
