# Supplementary material for: Effects of teaching experience and culture on choral directors’ descriptions of choral tone
Source: PLoS One. 2021 Dec 7;16(12):e0256587. doi: 10.1371/journal.pone.0256587 (PMC8651130; doi:10.1371/journal.pone.0256587)
Supplement: S4 Table — (DOCX) [file pone.0256587.s004.docx]

*S4 Table. Logistic regression for nonwestern choral selections*

|  | Coeff. | Std.Err. |
| --- | --- | --- |
| Healthy | -1.003863*** | 0.127 |
| Appropriate | .8954229*** | 0.122 |
| Constant | 0.200 | 0.330 |
| N. of cases | 1000 |  |
| * p<0.05, ** p<0.01,*** p<0.001 | |  |
